# Coral Pathogens Identified for White Syndrome (WS) Epizootics in the Indo-Pacific

**DOI:** 10.1371/journal.pone.0002393

**Published:** 2008-06-18

**Authors:** Meir Sussman, Bette L. Willis, Steven Victor, David G. Bourne

**Affiliations:** 1 ARC Centre of Excellence for Coral Reef Studies, School of Marine and Tropical Biology, James Cook University, Townsville, Australia; 2 Australian Institute of Marine Science (AIMS), PMB3, Townsville MC, Townsville, Australia; 3 Palau International Coral Reef Center (PICRC), Koror, Republic of Palau; Centre for DNA Fingerprinting and Diagnostics, India

## Abstract

**Background:**

White Syndrome (WS), a general term for scleractinian coral diseases with acute signs of advancing tissue lesions often resulting in total colony mortality, has been reported from numerous locations throughout the Indo-Pacific, constituting a growing threat to coral reef ecosystems.

**Methodology/Principal Findings:**

Bacterial isolates were obtained from corals displaying disease signs at three WS outbreak sites: Nikko Bay in the Republic of Palau, Nelly Bay in the central Great Barrier Reef (GBR) and Majuro Atoll in the Republic of the Marshall Islands, and used in laboratory-based infection trials to satisfy Henle-Koch's postulates, Evan's rules and Hill's criteria for establishing causality. Infected colonies produced similar signs to those observed in the field following exposure to bacterial concentrations of 1×10^6^ cells ml^−1^. Phylogenetic 16S rRNA gene analysis demonstrated that all six pathogens identified in this study were members of the γ-*Proteobacteria* family *Vibrionacae*, each with greater than 98% sequence identity with the previously characterized coral bleaching pathogen *Vibrio coralliilyticus*. Screening for proteolytic activity of more than 150 coral derived bacterial isolates by a biochemical assay and specific primers for a *Vibrio* family zinc-metalloprotease demonstrated a significant association between the presence of isolates capable of proteolytic activity and observed disease signs.

**Conclusion/Significance:**

This is the first study to provide evidence for the involvement of a unique taxonomic group of bacterial pathogens in the aetiology of Indo-Pacific coral diseases affecting multiple coral species at multiple locations. Results from this study strongly suggest the need for further investigation of bacterial proteolytic enzymes as possible virulence factors involved in *Vibrio* associated acute coral infections.

## Introduction

Reports on coral disease continue to rise [1] with currently 29 reported syndromes in the Caribbean [2] and 7 syndromes reported from the Indo-Pacific [3]. However, the causes for coral disease and the methods by which to investigate them are still heavily debated [4]–[6]. Most efforts are directed towards traditional surveillance [7], with comparatively less research directed towards developing strategies for active engagement in coral reef health management, disease prevention and cure [8]–[10]. Unfortunately, a lack of knowledge of coral disease causative agents propels this debate to a stand still. To date, only 5 bacterial species and one fungal agent have been determined as causative agents for coral infectious diseases [11]–[17], and currently no diagnostic tools or management efforts are able to validate these findings at a level required for active intervention. [18]–[19].

The study of disease in complex environmental settings is often difficult. Modern studies have cast a shadow on traditional culturing methods that are required to satisfy Henle-Koch's postulates [20], namely that a putative pathogen is first isolated on growth medium and then used in pure culture to duplicate disease signs in laboratory controlled infections. In many cases, more than 200 years after Henle-Koch's own revolution, these experiments often fail, requiring the introduction of modern rules and criteria in order to establish disease causation [21]–[22]. These are often based on statistical associations rather than on “cause and effect”.

Most microorganisms cannot be easily cultured [23] and other disease components, namely host susceptibility and environmental factors may jointly contribute to successful infections in what is known as the “disease triad” [24]. To this end, modern diagnostic tools have been developed that can be applied to enhance our knowledge of coral disease without targeting either a single or a cultivable agent. These tools include cloning and denaturing gradient gel electrophoresis [25], fluorescent *in situ* hybridization [26], microarrays [27] and metagenomics [28]–[30], just to name a few, and are used to either detect new pathogens or validate their presence once detected. Nevertheless, the benefits from isolating and culturing pathogens are still many, especially when precise disease identification for health control purposes is needed [31].

The study of epidemiology has revolutionized many concepts associated with disease studies [32] including some of the terminology used in infectious disease classifications. Traditional distinctions between primary vs. secondary, exogenous vs. endogenous and opportunistic agents [33]–[34] are being replaced by schemes classifying the genes involved in infectivity (the ability to physically infect a host [35]) and virulence (the severity of disease outcome inflicted by infection [36]). Modern studies have demonstrated that host, pathogen and environment form a constantly evolving disease equilibrium [37] contributing to a growing list of newly emerging infectious diseases [38]. The hierarchy of causation has been translated into causal models and complex outbreaks are now considered as multi-factorial, comprised of an often-unknown range of component causes [32], which need to be explored both independently and in conjunction with other causes. Nevertheless it remains a paradox, that despite the growing complexity in our understanding of disease causation, it is often expected that emerging infectious outbreaks be successfully curtailed before causation is fully established [39], shifting the focus from cure of individuals to disease-prevention in entire populations.

The aims of this study were therefore twofold: firstly, to identify possible causative agents for white syndromes widespread throughout the Indo-Pacific by combining both traditional microbial tools such as culturing with biochemical and molecular methods, and secondly, to investigate the aetiology of WS in order to recommend the development of novel diagnostic tools that could be implemented and validated in an active coral reef health management plan targeted “to protect against disease in the framework of the concept of ecosystem management” [40].

Since 2003, a variety of white syndromes have been reported from numerous locations throughout the Indo-Pacific and under various names [3], [41]–[44]. Willis et al. [3] suggested the use of a common term: white syndrome (WS), for Indo-Pacific scleractinian coral diseases displaying acute tissue loss exposing white skeleton in the absence of other disease signs or established causation. Three independent WS outbreaks were chosen for this 3-year study (2003–2006) in order to determine whether WS is one disease or possibly many, and whether a standard disease investigation protocol could be developed that could be used in future monitoring and management efforts (for a short video clip of a WS outbreak in the Republic of the Marshall Islands see Movie S1 in Supporting Information).

## Results

### Higher bacterial counts on WS corals

**Figure 1:**
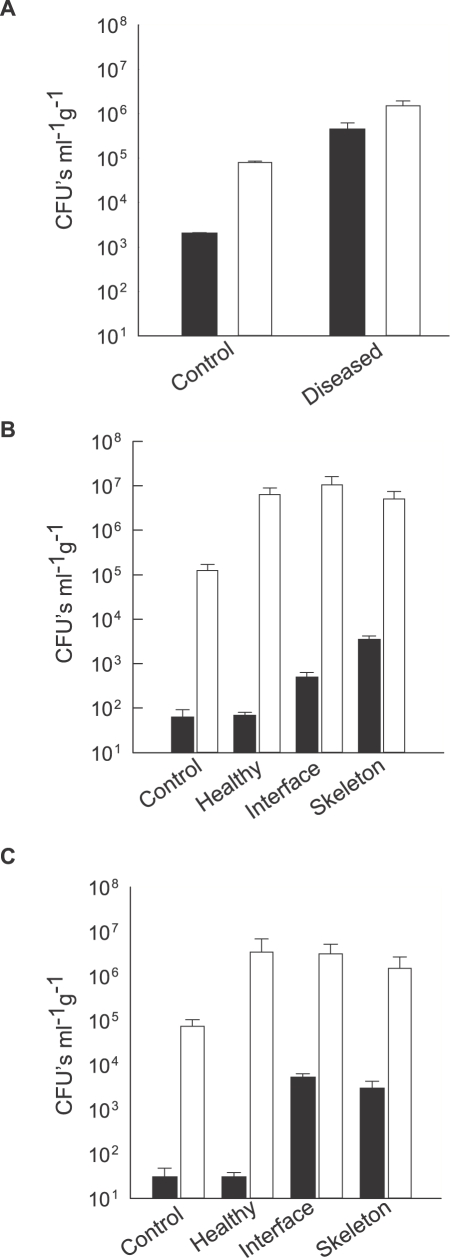
Bacterial density on corals sampled from the field: A. Mean CFU's ml^−1^ g^−1^ from crushed *Pachyseris speciosa* fragments sampled in Nikko Bay Palau. B. Mean CFU's ml^−1^ g^−1^ from crushed *Montipora aequituberculata* fragments sampled in Nelly Bay GBR C. Mean CFU's ml^−1^ g^−1^ from crushed *Acropora cytheria* fragments sampled in Majuro Atoll the Marshall Islands. ▪–Bacterial isolates streaked on TBCS agar. □-Bacterial isolates streaked on MA. Control–samples from coral fragments lacking disease signs. Healthy–Coral tissue lacking disease signs sampled from fragments displaying signs of disease. Interface–Coral tissue sampled at the border between exposed skeleton and healthy tissue. Skeleton–Exposed skeleton in areas of tissue lesions. CFU's ml^−1^ g^−1^ are presented in a logarithmic scale. Bars = Standard Errors.

Densities of cultivable bacteria (measured as CFU's ml^−1^ g^−1^ wet weight) associated with corals sampled from each of the three Indo-Pacific outbreak sites examined in this study were significantly higher on corals displaying disease signs than on those lacking disease signs (Fig. 1A–C). Mean CFU's from *Pachyseris speciosa* samples collected from Nikko Bay Palau (Fig. 1A) plated on a general heterotrophic Marine Agar (MA) were ∼20 times higher for diseased corals (Mean 1.50±0.42×10^6^ CFU's ml^−1^ g^−1^) than for corresponding samples lacking disease signs (Mean 8.0±0.5×10^4^ CFU's ml^−1^ g^−1^). A ∼200 fold difference was observed when the same samples were plated on TCBS agar selective for members of the family *Vibrionacae* (Mean 4.42±1.84×10^5^ and mean 2.0±0.1×10^3^ CFU's ml^−1^ g^−1^, respectively), suggesting higher *Vibrio* densities on diseased corals.

Cultivable bacterial densities were also found to be significantly higher on *Montipora aequituberculata* fragments (Nelly Bay GBR) displaying visual WS disease lesions, compared to coral fragments lacking lesions. Diseased fragments sampled from the interface (I) between lesions and healthy tissue (Fig 1B), gave rise to ∼7 times more *Vibrio* CFU's counts (Mean 4.92±1.53×10^2^ CFU's ml^−1^ g^−1^) than the corresponding healthy fragments (H) from the same corals (Mean 6.8±1.3×10^1^ CFU's ml^−1^ g^−1^). Fragments sampled from exposed coral skeleton (S) gave rise to ∼50 times more CFU's (Mean 3.42±0.77×10^3^ CFU's ml^−1^ g^−1^) than healthy fragments (H) from the corresponding corals. Fragments sampled from *Acropora cytherea* corals (Marshall Islands) similarly had a significantly higher mean CFU's counts on TCBS for samples derived from the lesion interface (I) and skeleton (S) compared directly against healthy looking fragments (H) of the corresponding corals (Fig. 1C), suggesting an association between *Vibrio* densities and disease lesions within a coral colony. Laboratory exposure trials were subsequently designed to test for isolate infectivity and to satisfy Hill's criterion 4 [22], namely that disease signs follow a “time sequence” with cause (bacterial presence) preceding effect (disease lesions).

### Inoculation Experiment I: Exposed colonies display disease signs

Bacterial strains isolated from corals displaying disease signs at each of the three outbreak sites (10 isolates from TCBS medium plates and 10 isolates from MA medium plates per site) were screened in infection trials with results from all inoculations presented in table 1. All five *P. speciosa* fragments (Nikko Bay Palau) inoculated with isolate P3 (1×10^6^ bacteria ml^−1^) developed disease signs following exposure for 96 h, while treatments with isolates P4 and P5 demonstrated lower infectivity (Fig. S1). Coral fragments in control treatments (n = 17) including treatments with 7 other TCBS derived isolates and 10 isolates from MA plates remained unaffected for the duration of the experiment. Healthy fragments of *M. aequituberculata* (Nelly Bay GBR) were only infected by one strain (P1) of the 20 strains tested, with 40% of fragments displaying disease signs after a 96 h exposure to P1. 100% and 12% of healthy *A. cytherea* fragments (Majuro Atoll Marshall Islands) exposed to strains P2 and P7, respectively, displayed disease signs after 36 h. A repeat of the experiment with strain P7 resulted in no further positive results and therefore the strain was eliminated as a possible putative pathogen. Results from inoculation experiment I satisfied Hill's criterion 4 [22] of “time sequence” (cause precedes effect) by demonstrating successful infectivity following putative pathogen inoculations.

**Table 1:** Inoculation experiment I

Outbreak	Nikko Bay Palau			Nelly Bay GBR	Majuro Atoll Marshall Islands	
**Experiment Duration (h)**	96			96	36	
**Inoculation Dose**	1×10^6^ cells ml^−1^			1×10^6^ cells ml^−1^	1×10^6^ cells ml^−1^	
**Coral host**	*Pachyseris speciosa*			*Montipora aequituberculata*	*Acropora cytherea*	
**Exposed (# inoculum treatments)**	20			20	20	
**Infected (# treatments producing lesions) ** 1	3			1	2	
**Strains causing infections ** 2	P3	P4	P5	P1	P2	P7 3
**Infectivity (%) ** 4. 5	100	60	40	40	100	12

1 The number of pure cultures in each experiment causing visible disease signs (lesions) on experimental fragments.

2 Bacterial isolates causing disease signs were named Pathogen 1–7 (P1–P7).

3 Inoculation experiment I was repeated for both isolates from the Marshall Islands (P2, P7) that demonstrated infectivity before ruling out isolate P7 as a possible putative pathogen.

4 Infectivity represents the percent of fragments (# exposed/# infected) within each of the treatments displaying visible disease signs (lesions)

5 No fragments in control treatments were infected

### Inoculation experiment II: Fulfilling Henle-Koch's postulates

**Figure 2:**
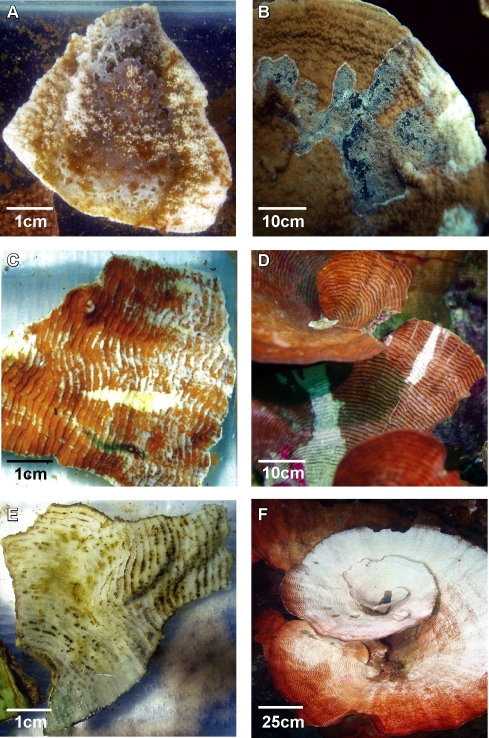
WS signs observed in the laboratory and in the field: A. *Montipora aequituberculata* exposed to pathogen P1 in laboratory inoculation experiment. B. *M. aequituberculata* with WS signs in the field (Nelly Bay GBR). C. *Pachyseris speciosa* exposed to pathogen P6 in laboratory inoculation experiment. D. *P. speciosa* with WS signs in the field (Nikko Bay Palau). E. *P. speciosa* exposed to pathogen P3 in laboratory inoculation experiment. F. *P. speciosa* with WS signs in the field (Nikko Bay Palau).

**Figure 3:**
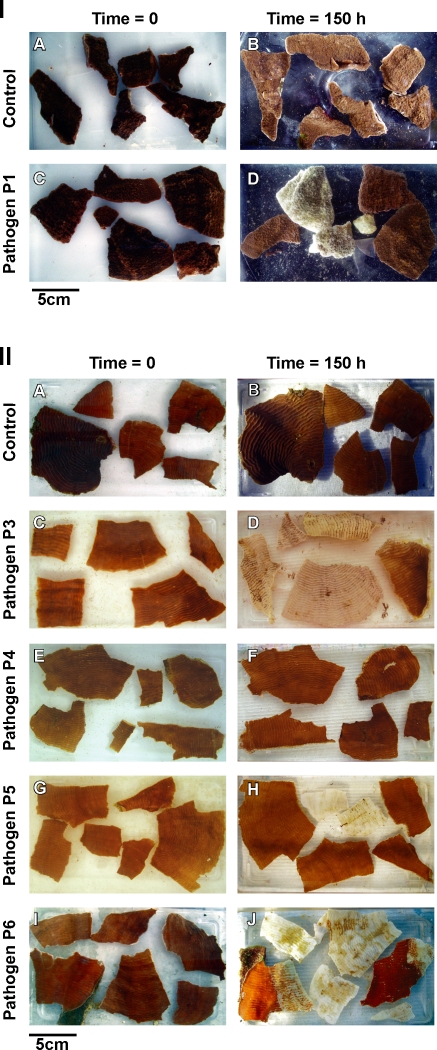
Inoculation experiment II: I A–B. *Montipora aequituberculata* coral fragments in un-inoculated control treatment (t = 0h and t = 150h). I C–D. *M. aequituberculata* coral fragments exposed to 1×10^6^ cells ml ^−1^ of culture P1 (t = 0h and t = 150h). II A–B. *Pachyseris speciosa* coral fragments in un-inoculated control treatment (t = 0h and t = 150h). II C–D. *P. speciosa* coral fragments exposed to 1×10^6^ cells ml^−1^ of culture P3 (t = 0h and t = 150h). II E–F. *P. speciosa* coral fragments exposed to 1×10^6^ cells ml^−1^ of culture P4 (t = 0h and t = 150h). II G–H. *P. speciosa* coral fragments exposed to 1×10^6^ cells ml^−1^ of culture P5 (t = 0h and t = 150h). II I–J. *P. speciosa* coral fragments exposed to 1×10^6^ cells ml^−1^ of culture P6 (t = 0h and t = 150h).

Results from three replicated experimental inoculation trials conducted to fulfil Henle-Koch's' postulates and determine the virulence of putative pathogens by causing mortality to infected corals are presented in table 2. Healthy colony fragments exposed to putative pathogens P1–P6 (1×10^6^ cells ml^−1^) displayed signs of disease similar to those observed in the field in all experiments (Fig. 2A–F). Exposure of *M. aequituberculata* to putative pathogen P1 resulted in lesions covered by a sulphurous deposit, which matched disease signs in the field (Fig 2A–B). Exposure of *P. speciosa* to putative pathogens P4 and P6 began by producing linear lesions resembling field observed lesions (Fig. 2C–D), while *P. speciosa* fragments exposed to P3 and P5 resulted in the development of larger lesions similar to a second, more common type of lesion observed at the site (Fig. 2E–F). Coral fragments inoculated with control strains (non-pathogenic) and un-inoculated control fragments did not develop signs of WS lesions (Fig. 3IA–B, 3IIA–B) in contrast to lesion signs and mortality observed in all treatments with putative pathogens (Fig 3IC–D, 3IIC–J). Bacterial isolates from infected fragments retrieved at the conclusion of the experimental exposure, demonstrated 100% 16S rRNA gene sequence identity to inoculated strains. Recovery of inoculated strains from infected fragments fulfilled Henle-Koch's postulates for all 6 proposed agents examined in this study.

**Table 2:** Inoculation experiment II

Outbreak	Nikko Bay Palau	Nelly Bay GBR	Majuro Atoll Marshall Islands
**Date**	2005	2003	2004
**Target host/s**	*Pachyseris speciosa*	*Montipora aequituberculata*	*Acropora hyacinthus*
**Putative pathogen**	P3, P4, P5, P6 1	P1	P2
**Effective concentration**	1×10^6^ cells ml^−1^	1×10^6^ cells ml^−1^	1×10^6^ cells ml^−1^
**Temperature in tanks**	29.9°C	28°C	30°C
**# fragments (n)**	195	360	80
**# inoculum treatments**	11	3	3
**# tanks per treatment (N)**	3	12	4
**# fragments per tank**	5	6	4
**Mean infected (Infectivity) ** 2	0.88±0.08	0.55±0.13	0.94±0.05
**P value**	P<0.00001	P = 0.00005	P<0.00001
**Mean mortality rate (Virulence) ** 3	0.66±0.05	0.87±0.09	0.66±0.11
**Mean pathogenicity ** 4	0.58±0.11	0.48±0.15	0.62±0.24
**Mean lesions on control fragments ** 5	0.04±0.02	0.08±0.06	None
**LT50 ** 6	60h	180h	18h

1 Data for separate inoculation experiments with isolates P3–P6 was pooled together.

2 Mean infectivity was calculated as mean #infected / # exposed±SE.

3 Mean mortality rate, or virulence, was calculated as mean # dead / # infected±SE.

4 Mean pathogenicity was calculated as mean # dead/ #exposed±SE.

5 Mean lesions on control fragments were calculated as mean # lesions / # controls±SE.

6 LT50 is the time needed to cause 50% mortality of the exposed fragments.

The proportion of exposed fragments per tank that became infected (infectivity) varied among the experiments, with 88% of fragments exposed to P3–P6, 55% of fragments exposed to P1 and 94% of fragments exposed to strain P2 becoming infected. Pathogenicity (proportion of exposed fragments that died) measured 58%, 48% and 62%, and mortality rate, or virulence (proportion of infected fragments that died) equalled 66%, 87% and 66% for putative pathogens from Palau, Magnetic Island and the Marshall Islands, respectively. Similarly, the times needed for 50% of the fragments to experience mortality (LT50) were 60h, 180h and 18h, respectively.

### Aetiology of WS: Adhesion of pathogens to coral tissue

**Figure 4:**
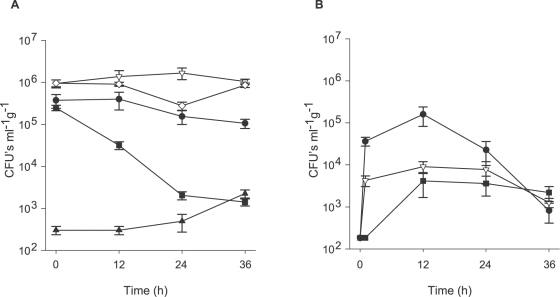
Adhesion experiment: A. CFU's counts (ml^−1^) from SW samples. •-1×10^6^ cells ml^−1^ of culture P1 inoculated into SW without corals. ⋄-1×10^6^ cells ml^−1^ of culture from control isolate MF1 inoculated into SW without corals. ▪-1×10^6^ cells ml^−1^ of culture P1 inoculated into SW with *Montipora aequituberculata* coral fragments. ▿-1×10^6^ cells ml^−1^ of culture MF1 inoculated into SW with *M. aequituberculata* coral fragments. ▴-SW with *M. aequituberculata* coral fragments without inoculation. B. CFU's counts (ml^−1^ g^−1^) from crushed coral fragments. •-1×10^6^ cells ml^−1^ of culture P1 inoculated into SW with *M. aequituberculata* coral fragments. ▿- 1×10^6^ cells ml^−1^ of culture MF1 inoculated into SW with *M. aequituberculata* coral fragments. ▪-*M. aequituberculata* coral fragments without inoculation. Time represents hours (h) following exposure.CFU's ml^−1^ g^−1^ are presented in a logarithmic scale. Bars = Standard errors.

Putative pathogen P1 (Nelly Bay GBR) demonstrated an 87% reduction in mean seawater CFU's (Fig 4A) within the first 12 h following inoculation into aquaria with fragments of *M. aequituberculata* (from mean 2.48±0.37×10^5^ cells ml^−1^ at t 0 to mean 3.17±0.67×10^4^ cells ml^−1^ at t 12). In comparison, only a 6% reduction was observed when the same corals were inoculated with control bacterial isolate MF1 (from mean 9.60±1.81×10^5^ cells ml^−1^ at t 0 to mean 9.07±1.01×10^5^ cells ml^−1^ at t 12). CFU's from un-inoculated control aquaria averaged 3.0±0.69×10^2^ cells ml^−1^ after 12 h. After 36 h, mean CFU counts from aquaria seawater treated with P1 dropped even further to 0.6% of the original inoculation concentration (Men 1.41±0.27×10^3^ cells ml^−1^), which was similar to the density of cells in control tanks (Mean 2.26±0.49×10^3^ cells ml^−1^). In contrast, putative pathogens that were inoculated into sterile seawater without corals maintained a constant density of viable counts in suspension throughout the experiment (Fig. 4A), eliminating the possibility that bacteria died from the seawater itself or may have settled on the sides or bottom of aquaria. *Vibrio* density in aquaria containing *M. aequituberculata* fragments, which were inoculated with non-pathogen MF1 remained unchanged after 36 h, with mean 1.04±0.15×10^6^ cell ml^−1^ (100%) retrieved on TCBS agar plates.

CFU counts of crushed coral samples (CFU ml^−1^ g^−1^ wet weight) from aquaria inoculated with P1 reached a mean of 1.60±0.78×10^5^ ml^−1^ g^−1^ after 12 h (Fig 4B). In comparison, fragments from aquaria inoculated with control bacteria (MF1), or un-inoculated controls, resulted in CFU counts that were 94% and 97% lower after 12 h (Mean 9.08±2.82×10^3^ ml^−1^ g^−1^ and mean 4.12±2.45×10^3^ ml^−1^ g^−1^, respectively). Table S1 summarizes the data from adhesion experiments conducted with putative pathogens and controls isolated from the three infection sites examined in this study.

### Loss of *Symbiodinium* followed by tissue lesions

**Figure 5:**
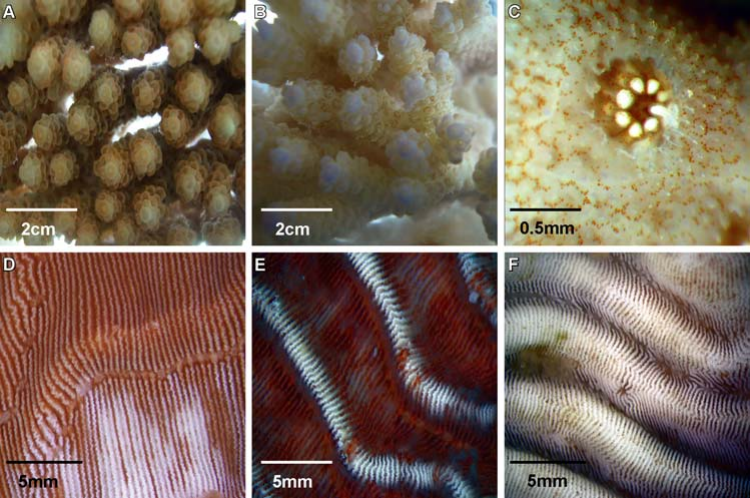
Disease progression: A. *Acropora hyacinthus* fragmennt inoculated with 1×10^6^ cells ml^−1^ of culture P2 (t = 0h). B. Loss of *Symbiodinium* from *A. hyacinthus* inoculated with 1×10^6^ cells ml^−1^ of culture P2 (t = 12h). C. Polyp and surrounding tissue-loss of *Symbiodinium* from *A. hyacinthus* inoculated with 1×10^6^ cells ml^−1^ of culture P2 (t = 12h). D. Loss of *Symbiodinium* cells from *Pachyseris speciosa* inoculated with 1×10^6^ cells ml^−1^ of culture P3 (t = 12h). E. Tissue lesions on *P. speciosa* inoculated with 1×10^6^ cells ml^−1^ of culture P3 (t = 24h). F. Exposed skeleton on *P. speciosa* inoculated with 1×10^6^ cells ml^−1^ of culture P3 (t = 60h).

Detailed photographs taken of *A. hyacinthus* fragments infected experimentally with P2 (Fig 5A–C) revealed 2 distinct disease-phases. An initial loss of *Symbiodinium*, visible as tissue paling was observed after 9–12 h of exposure (Fig 5B–C) followed by developing tissue lesions. Similar patterns of paling were also observed when *P. speciosa* fragments were exposed to P3 (Fig 5D). Paling and loss of *Symbiodinium* commenced in coenosarc tissue (tissue between polyps) in distinct linear patterns starting 12 h post inoculation and corresponding with the peak in viable CFU counts retrieved from coral tissue. These early signs of disease then developed into lesions that resembled those observed in the field (Fig 5E–F), suggesting that disease progression was consistent (Hill's [22] criterion 2) and followed measurable steps (Evans' Rule F [21]). For a 24h time lapse video clip of *A. hyacinthus* inoculated with pathogen P2, see Supporting Information Movie S2.

**Figure 6:**
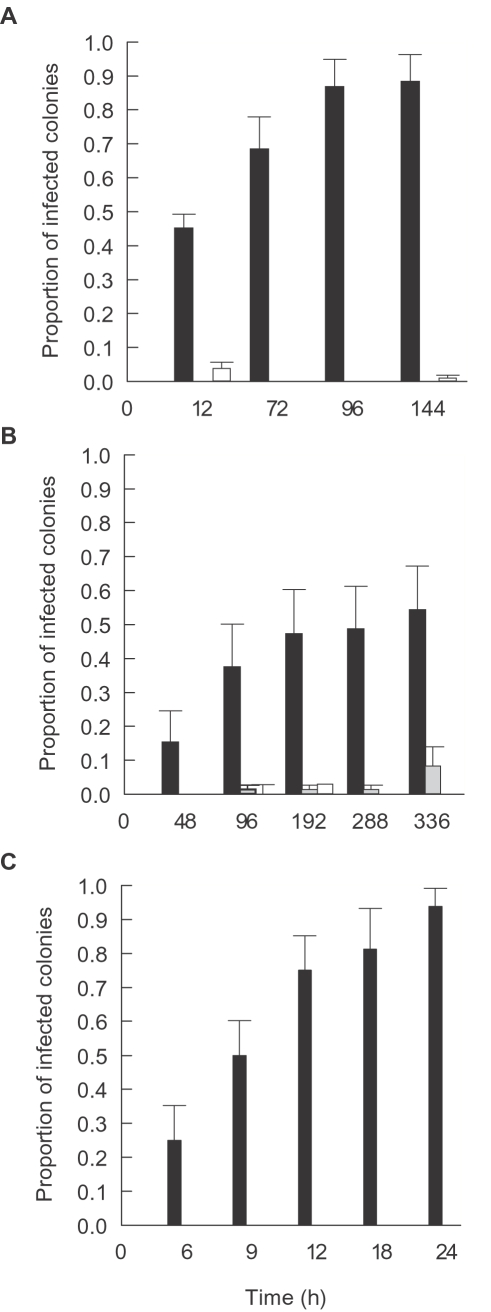
Disease transmission: A. vMean proportion of infected *Pachyseris speciosa* coral fragments displaying WS signs following exposure to cultures of P3–P6 in comparison to proportions in inoculated and un-inoculated control treatments. B. Mean proportion of infected *Montipora aequituberculata* coral fragments displaying WS signs following exposure to culture of P1 in comparison to proportions in inoculated and un-inoculated control treatments. C. Mean proportion of infected *Acropora cytherea* coral fragments displaying WS signs following exposure to culture P2 in comparison to proportions in inoculated and un-inoculated control treatments. ▪-Coral fragments inoculated with 1×10^6^ cells ml^−1^ of putative pathogen cultures. 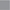-Coral fragments inoculated with 1×10^6^ cells ml^−1^ culture of non-pathogen isolates. □-Coral fragments without inoculation. Time represents hours (h) following exposure. Bars = Standard errors.

In all experimental treatments inoculated with putative pathogens P1–P6, the proportion of fragments displaying acute disease signs (lesions) increased with time to between 55% and 94% of fragments per tank (Fig 6A–C) conforming with Evans' rules D and E [19], namely that disease occurs, temporally, following specific incubation times and that the number of new cases and the severity of outcome should correlate positively with time. The proportion of *P. speciosa* fragments from Palau, *M. aequituberculata* fragments from Nelly Bay and *A. hyacinthus* fragments from the Marshall Islands displaying acute disease signs increased consistently and significantly within the first 96 hours (Fig. 6A–6B) and 12 hours (Fig. 6C) of the start of inoculation experiment II, at each site, respectively, resembling standard infection curves [32]. In contrast, 0–8% of fragments in inoculated and un-inoculated control treatments developed disease signs (Fig 6A–C).

### Isolates associated with disease signs are proteolytically active

Isolated bacteria (152 strains) recovered from both diseased and healthy corals were screened for proteolytic activity using the asocasein assay and specific PCR primers targeting the zinc-binding site of a *Vibrio* family zinc-metalloprotease. A total of 48% of strains (n = 33 strains) retrieved from diseased *P. speciosa* in the field (Nikko Bay Palau) demonstrated high (≥3U) or medium (1-3U) proteolytic activity compared with 30% strains (n = 23 strains) demonstrating high or medium activity that were retrieved from non-diseased colony fragments sampled in the field (Table 3). This difference, however, was not found to be statistically significant (Pearson's χ^2^ = 1.825, DF = 1, p = 0.177). In contrast, 11 positive PCR bands and derived partial sequences of the *Vibrio* zinc-metalloprotease gene were obtained from DNA of isolates retrieved from diseased *P. speciosa* sampled in the field compared with only 1 partial sequence from a non-diseased colony fragment. This difference was found to be significant by testing for Pearson's chi-square (χ^2^ = 6.763, DF = 1, p = 0.0093).

**Table 3:** Proteolytic activity of bacterial isolates (Nikko Bay Palau)

	Bacterial isolates retrieved from field *Pachyseris speciosa* 1		Total
	Diseased colonies	Non-diseased colonies	
**+ve PCR product ** 2	11	1	12
**−ve PCR product ** 2	22	22	44
**Total**	33	23	56
**High proteolytic activity ** 3	6	4	10
**Medium proteolytic activity ** 4	10	3	13
**No proteolytic activity ** 5	17	16	33
**Total**	33	23	56

1 Isolates retrieved from diseased and non-diseased *Pachyseris speciosa* colonies sampled in Nikko Bay Palau .

2 Specific amplification of *Vibrio* zinc-metalloprotease active zinc binding site.

3 High proteolytic activity >3U measured by the asocasein assay.

4 Medium proteolytic activity 1-3U measured by the ascasein assay.

5 No proteolytic activity <1U measured by the asocasein assay.

Similar results were obtained by screening field isolates from Nelly Bay GBR (Table S2). Bacteria demonstrating high and medium proteolytic activity by the asocasein assay made up 70% of all isolates retrieved from coral skeletons (S) exposed by WS disease at Nelly Bay GBR and 57% of all isolates from the lesion interfaces (I), compared with only 24% of all isolates obtained from healthy (H) tissue fragments on diseased colonies, demonstrating a significant difference in proteolytic activity between isolates associated with disease signs (I+S) and healthy (H) tissue (Pearson's χ^2^ = 6.446, DF = 1, p = 0.011). A significant difference was also obtained for the same 38 isolates when screened by the molecular method using PCR primers (Pearson's χ^2^ = 12.518, DF = 1, p<0.0001). Finally, screenings by the molecular method performed on DNA extracted from 56 isolates retrieved from both infected and non-infected fragments at the conclusion of inoculation experiment II in Palau (Table S3), demonstrated that results obtained by screening field isolates were consistent with screening laboratory derived isolates (Pearson's χ^2^ = 6.725, DF = 1, p = 0.010). Thus, in both field and laboratory infections, the presence of a *Vibrio* family zinc-metalloprotease was associated with disease signs conforming to Evans' rules B, C and G [21], suggesting that bacterial proteolytic activity may cause or contribute to observed WS lesions.

### Pathogens identified by this study form a taxonomic cluster

**Figure 7:**
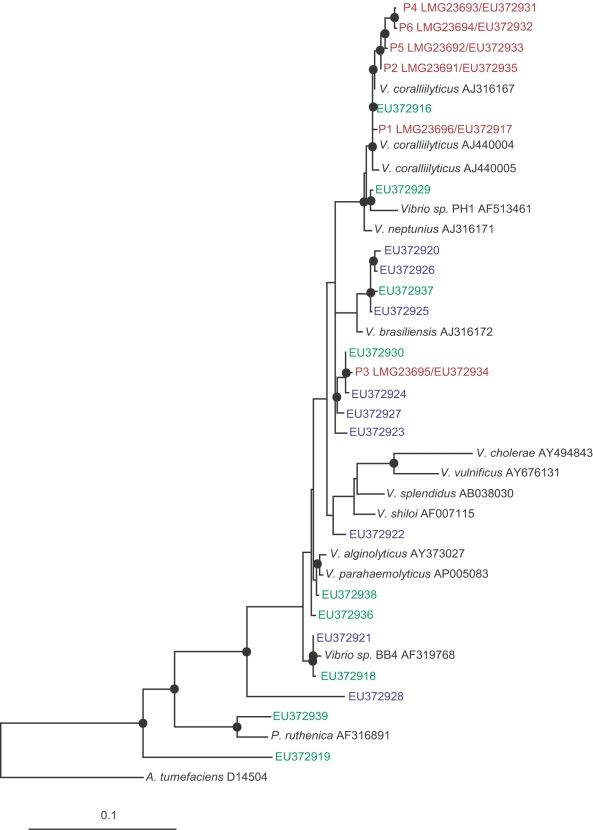
Phylogenetic tree of proteolitically-active isolates: Evolutionary distance maximum likelihood analysis based on 16S rRNA gene sequences of isolates obtained by this study. Coral pathogens are marked in red. Reference strains are marked in black. Isolates that demonstrated high proteolytic activity (asocasein assay) and tested positive for a zinc-metalloprotease gene are presented in blue (Palau isolates) and in green (Nelly Bay GBR isolates). Nodes represent bootstrap values ≥50% based on 1000 re-samplings. Scale bar corresponds to 10% estimated sequence divergence.

Based on near complete 16S rRNA gene sequence comparisons, the six pathogens clustered in a tight taxonomic group and were found to share between 98–99% sequence identities with the previously characterized coral-bleaching pathogen *Vibrio coralliilyticus* [14]. All isolates which tested positive for the zinc-metalloprotease zinc-binding site and exhibited high proteolytic activity (when screened by the asocasein assay) were used to construct a maximum likelihood phylogenetic tree based on their 16S rRNA gene (Fig. 7). Our findings demonstrate that more isolates possess the genetic capacity to become proteolytically active than the six coral pathogens identified in this study, suggesting that successful infections require the expression of additional virulence genes, but also that other non-pathogens might be indirectly involved in enhancing infections.

## Discussion

This study reports the successful isolation and identification of bacterial infectious agents implicated in a group of widespread Indo-Pacific coral diseases that affect numerous species at various geographical locations. Six coral pathogens were identified with close 16S rRNA gene phylogenetic affiliation with the previously identified coral pathogen *V. coralliilyticus* [14]. *Vibrio* pathogens have been previously demonstrated to cause fish, eel, shrimp and human mortalities [45]–[49]. Seasonal bleaching of the coral *Oculina patagonica* in the Mediterranean Sea has been shown to be caused by *V. shiloi* [*5]*, [11], [50]–[*51*] and *V. coralliilyticus* has been identified as the aetiological agent of *Pocillopora damicornis* bleaching in the Indian Ocean [14], [52]–[53]. Other coral diseases in the Caribbean, such as White Band Disease type II, Yellow Blotch/Band and Dark Spots Disease, were found to be associated with elevated *Vibrio spp*. prevalence [4], [54]–[56], suggesting the involvement of *Vibrio* strains in numerous coral diseases including coral bleaching [5], [57]–[59].

This study has found high prevalence of *Vibrio spp*. to be associated with WS signs in all diseased colonies sampled from three WS outbreaks. An association between bacterial presence and disease signs does not provide proof that bacteria actually cause the disease. However, such an association already fulfils requirements put forth by Evans' rule A [21], namely that the “prevalence of the disease should be significantly higher in those exposed to the putative cause than in cases controls not so exposed”. It also complies with the Read [60] definition of virulence, which highlights an agent's contribution to reduction in host fitness caused by exploitation.

When bacterial strains were inoculated into tanks with healthy coral fragments, only putative pathogens adhered to coral tissue and a consistent peak in cultivable *Vibrio* abundance, 500–1000 fold greater than was found for control strains, was observed on fragments exposed to putative pathogens 12 h post inoculation. This experiment confirmed Hill's criterion of “time sequence” [22], suggesting that following exposure and prior to the development of visual disease signs (lesions), putative pathogens were able to migrate towards the coral fragments, adhere to coral tissue and survive initial contact in a viable state. In contrast, control strains were unable to perform this transmission, suggesting that motility towards corals and adhesion may be regarded as traits involved in pathogenicity. Initial visual signs of tissue paling and lesions were observed following a peak in cultivable bacterial abundance for all six putative pathogens, demonstrating a common aetiology of adhesion followed by disease progression. Despite this peak in cultivable *Vibrio* abundance 12 h post exposure, less than 1% of the original inoculation was retrieved by plating coral fragments, potentially indicating that *Vibrio* cells entered a viable but non-culturable (VBNC) state [61], or alternatively, died.

The colonization of target hosts by *Vibrio* pathogens has been studied in detail, particularly the ability of *Vibrios* to adhere to mucus found either inside the gastro- internal track or externally on fish or corals [62]–[63]. Denkin and Nelson [64] have demonstrated that the transcription of zinc-metalloprotease by the fish pathogen *V*. *anguillarum* is regulated by mucus and can only occur after adhesion is completed. This duality in *Vibrio* function is often referred to as the “transmission-virulence trade-off” [65] and highlights the fact that the ultimate goal of *Vibrio* pathogenicity is not to kill a host, or to complete a necessary biological life-cycle within it, but to re-enter the environment in larger numbers and initiate a new cycle of infections [66]. It explains why *Vibrio* pathogens are commonly found in environmental reservoirs [26], or transmitting through the water column, like pathogen P6 isolated in this study from seawater above infected corals at Nikko Bay Palau.

This study has demonstrated that 55%–94% of coral fragments exposed to pathogens cultured from diseased corals at their respective field sites become infected and that 66%–87% of those infected die, compared with significantly lower infection and mortality for fragments exposed to control bacterial strains (0–8%). These results conform to Evans' rule G [21], requiring that experimental reproduction of the disease should occur in higher incidence in those exposed to the putative cause than in those not so exposed. However, a proportion of exposed fragments did not develop disease signs, demonstrating that the probability of becoming infected may not be equal among healthy colony fragments collected from the field, and that other host related factors potentially contribute to successful infections. Such unknown factors can be explored in future inoculation trials.

The presence of *Vibrio spp*. on both healthy and diseased corals has led to the conclusion by some authors [67]–[68] that *Vibrio* infections of corals may be opportunistic in nature. This assumption fits well into models of disease occurring in environmental settings, where multiple factors, such as host density [69] and temperature [70] have been shown to influence the probability of successful infections. Combinations of virulent and a-virulent *Vibrio* strains are found readily in environmental samples [71] with non-clinical *V. cholera* strains found to be capable of causing infections despite lacking the cholera toxin gene [72]. Many *Vibrios* specialize in multiple host attachment and detachment [73]–[75], suggesting a broad scope for potential coral infections by *Vibrios* including possible host shifts due to fish depletion from coral reefs [76]. Amaro and Biosca [46] have demonstrated that *Vibrio vulnificus* biotype 2 is both a primary pathogen for eels and an opportunistic pathogen for humans, indicating that the identification of opportunistic pathogens requires rigorous testing. Nevertheless, none of the claims to define *Vibrio* coral infections as opportunistic have so far provided conclusive evidence to show that suspects (identified by molecular screening methods) found on healthy corals are in fact pathogenic (whether opportunistic or not), or that only compromised hosts become infected. In addition, not all coral mortalities are caused by infectious agents, but rather by exposure to extreme conditions, such as pesticides or high nutrient levels [77]–[78], which may result in indirect shifts in microbial abundance. Infectious outbreaks can be distinguished from non-infectious ones by plotting infection curves [32] to demonstrate a bell-shape increase and decrease in incidence rate with time.

This study did not find evidence for the presence of coral pathogens on healthy corals in the field, nor evidence that exposed fragments might be successfully infected due to stress other than the direct exposure to the pathogens themselves. Control treatments in all inoculations remained healthy, including a proportion of those exposed to pathogens. Further studies are recommended to determine the prevalence of pathogens in field samples by developing diagnostic tools to target specific virulence genes in large scale screening efforts. These studies could then determine the proportion of exposed corals in the field that develop acute disease signs and should become an integral part of establishing acute vs. chronic disease prevalence in environmental studies.

This is the first study to diagnose proteolytic activity as a possible component of the aetiology of WS through the screening of more than 150 isolates from both diseased and non-diseased corals. Zinc-metalloproteases have been characterized as virulence factors in many *Vibrio* family pathogens, such as *V. cholera* [79], *V. vulnificus* [80], *V. harveyi* [81] and *V. anguillarum* [82]. *Vibrio* zinc-metalloproteases are involved in cleavage of connective tissue [83], para-cellular perturbation [84], swarming and adhesion to mucus [85] and detachment [86]. The coral bleaching pathogens *V. shiloi* and *V. coralliilyticus* have been previously shown to harbour a zinc-metalloprotease [53], [87] along with other toxins that cause photosynthetic 5 inhibition of coral *Symbiodinium* [88]. *Serratia marcescens*, the aetiological agent of acroporid serratiosis (coral White Pox disease [13]), resulting in acute tissue lesions, also possesses a virulent zinc-metalloprotease capable of connective tissue degradation [89]. However, it has been shown that both clinical and non-clinical strains possess zinc-metalloprotease genes [90], suggesting that it may not be the only virulence factor to cause successful infections. This study provided similar results, underlining the need to search for additional virulence factors in future studies.

Recent studies by Ainsworth et al. [91] did not detect bacteria associated with WS lesions of diseased corals sampled at Heron Island on the GBR, using direct microscopic techniques. In contrast, samples of WS corals obtained from Heron Island in this study for screening purposes demonstrated an abundance of *Vibrio* spp. isolates on WS lesions, including proposed putative pathogens that are proteolytically active and possess a zinc-metalloprotease gene. These contradicting findings underline the importance of ‘comparative validation’ [92] in disease research and the need for standardized protocols for disease detection using better diagnostic tools.

Further histopathological studies by Ainsworth et al. [68], [91] utilizing commercial labelling kits have found that coral fragments displaying WS signs test positive for DNA fragmentation. These observations led to the hypothesis that WS is potentially the result of coral programmed cell death. However, further proof is needed in order establish whether DNA fragmentation (or apoptosis) in corals is cause or effect. The induction of apoptosis by bacterial pathogens (*Salmonella Sp., E. coli, Shigella sp., C. difficile, L. monocytogenes, C. parvum* and others) has been previously demonstrated by many studies [93]-[97], suggesting a possible link between bacterial infections and apoptosis. This link can be tested in future pathogen-exposure trials and used to design novel diagnostic protocols for WS, which would target bacterial enzymes causing DNA fragmentation.

In summary, this study demonstrated consistent results in applying cost effective culturing techniques combined with biochemical and molecular tools towards successful pathogen isolation, coral disease investigation and sample screening. Future research should be conducted to explore the virulence components of all six pathogens identified in this study and to test the contribution of multiple factors (pathogen, environment and host related) to the aetiology of WS. Enhanced monitoring and management of WS outbreaks will not only benefit coral health, but would also further validate results obtained in this study.

## Materials and Methods

### Isolation and growth of bacteria from coral samples

For inoculation experiment I, ten fragments (2–10 g wet weight) from corals displaying WS disease signs and ten fragments (2–10 g wet weight) from corals lacking WS disease signs were collected from depths between 3–15 m at each of the following locations: 1) Nelly Bay fringing reef (S19 10′ E 146 52′) at Magnetic Island in the central section of the Great Barrier Reef (GBR) in September 2003; 2) Majuro Atoll the Republic of the Marshall Islands (N 9 00′ E 168 00′) in August 2004; and 3) Nikko Bay, an enclosed bay among rock islands in the Republic of Palau (N 7 30′ E 134 30′) in February 2005. WS mainly affected plate colonies of *Pachyseris speciosa* in Palau, tabular species of *Acropora* (*A. cytherea*, *A. hyacinthus* and *A. clathrata*) in the Marshall Islands and plate colonies of *Montipora aequituberculata* at Nelly Bay GBR. At each site, samples were transported from the reef to the laboratory in sterile containers.

For calculating the abundance of bacteria associated with diseased and non-diseased fragments, the following sub-samples were obtained at each site: healthy tissue from coral fragments with no disease (CON, n = 3); tissue adjacent to lesions on coral fragments with WS disease signs (INF, n = 3); healthy tissue on coral fragments displaying disease signs (H, n = 3); lesion interface on coral fragments displaying disease signs (I, n = 3); and exposed coral skeleton on coral fragments displaying disease signs (S, n = 3).

Samples were crushed and diluted with 10 ml of 0.22 µm filtered seawater (Millipore, USA), and then vortexed for 3 min at maximum speed before being left to settle for 3 min [26]. Supernatant (100 µL) was streaked on agar plates containing a general heterotrophic bacterial medium (Marine Agar: 1.8% Marine Broth, Difco-2216, USA 0.9% NaCl, 1.8% Agar Bacto, Difco-214010, USA) and thiosulfate citrate bile salts sucrose (TCBS) agar, a *Vibrionacea* selective growth medium (Difco, USA). Plating was conducted in triplicates of serial dilutions (1×10^−1^–1×10^−6^) followed by incubation overnight at 30°C. Cultivable strains were quantified by counting colony forming units (CFU's) and the density of bacteria associated with corals was determined as mean CFU's per 1 ml of crush derived from 1 g (wet weight) of coral tissue (CFU’s ml^−1^ g^−1^). Single CFU's were picked from both Marine Agar (MA) and TCBS plates and transferred to fresh MA plates for further analyses.

Single isolates were grown in 250ml sterile flasks containing sterile marine broth (MB) media incubated at 30 °C for 18h (i.e. to end of the logarithmic phase) with constant shaking (150rpm). Cell density in pure cultures was determined by plating triplicates of serial dilutions on MA and by measuring absorbance (595nm) in sterile microtitre well plates (n = 6).

Additional bacterial isolates from fragments displaying signs of ongoing tissue loss in association with WS and from healthy fragments (controls) were retrieved for screening purposes from corals at Heron Island, GBR (March 2004) and at Dip Reef GBR (November 2004).

### DNA extraction, PCR amplification and gene sequencing

Genomic DNA extraction from pure cultures of bacterial isolates retrieved by this study was performed using the Wizard genomic DNA purification kit (Promega, USA) as per the manufacturer's instructions. The 16S rRNA gene was amplified by using universal primers 27F and 1492R. [98]. In addition, primers HA-F (5′ –CATGAGGTCAGCCACGGTTTTACTGAGCAG) and HA-R (5′–CGCGCGGTTAAACACGCCACTCGAATGGTGAAC (Invitrogen, NZ) targeting a ∼225 bp region including the zinc binding site of *Vibrio*-family zinc-metalloproteasses [99] were used to screen all bacterial genomic DNA. PCR reactions (50 µL) were run on an Eppendorf Mastercycler with the reaction mix consisting of 10 pmol of each primer, 5 µL of 10xPCR buffer with 15 mM MgCl_2_, 50 nmol dNTP, 10 ng template DNA and 1U Taq (iTaq, Intron Biotechnology, Korea). DDW (Milli-Q, millipore) was added to the volume of 50 µL. Cycling conditions consisted of: 1) 27F/1492R-a 5 min denaturation step at 94°C followed by 30 cycles of 1 min at 94°C, 1 min at 52°C and 1 min at 72°C and concluded by a 7 min extension step at 72°C; and 2) HA-F/HA-R-a 5 min denaturation step at 94°C followed by 30 cycles of 20 sec at 94°C, 20 sec at 55°C and 1 min at 72°C, concluded by a 5 min extension step at 72°C. Amplified bands of the correct size were confirmed on a 1% ethidium bromide stained TAE agarose gel and amplified gene products were sequenced at MACROGEN Inc. (Seoul, Korea) on an ABI PRISM 3730XL analyzer (96 capillary-Applied Biosystems, CA, USA) using the ABI PRISM BigDyeTM Terminator Cycle Sequencing Kit. Retrieved gene sequences were aligned for closest matches using BLAST [100]. In total, 152 partial sequences were retrieved from coral fragments displaying WS signs and from controls sampled at the three-infection sites.

### Phylogenetic analyses

Sequences were checked for chimera formation with the CHECK_CHIMERA software of the Ribosomal Database Project [101]. Sequence data were aligned to the most similar sequence using the BLAST database algorithm [100], and then further analysed with the ARB software package [102]. Tree topologies were evaluated by reconstructing phylogenies using maximum likelihood evolutionary distance analysis (Phylip Distance Method with Jukes and Cantor model) of aligned near full-length sequences (>1200 bp). Regions of ambiguous sequence (N) were removed from the analysis. Bootstrap values were obtained for branching patterns using the Phylip software package (version 3.65 [103]) and values ≥50% were included for main nodes of the tree.

### Infection experiments

Infection experiments were run as incurred matrices in 2 consecutive stages, described below as inoculation experiments I and II.

### Inoculation experiment I: Testing for infectivity of bacterial isolates

To screen bacteria for infectivity (the ability to initiate visual disease signs (lesions) regardless of their severity [35]), 20 isolates retrieved from coral samples at each of the three sites (10 most abundant isolates on both MA and TCBS plates from both healthy and diseased colonies at each site) were grown to end logarithmic phase in MB (as described above) and inoculated individually into 7L sterile aerated tanks (final inoculum concentration = 1×10^6^ cells ml^−1^) containing 4–6 healthy fragments of corals collected from sites without disease signs (i.e. healthy fragments of *Pachyseris speciosa* from a healthy Palau site, healthy *Acropora cytherea* fragments from a healthy Marshall Islands site, and healthy *Montipora aequituberculata* fragments from a healthy Nelly Bay site). Prior to bacterial inoculation, coral fragments were acclimatized for 5 days to allow recovery from handling and fragmentation following a protocol by Kushmaro et al. [50]. Each of the 20 culture inoculations was tested in two tanks [n = 168–252 fragments per site, N = 21 inoculation treatments including 1 negative control treatment]. The negative control tanks contained coral fragments with no bacteria added. Seawater in the tanks was replaced every 48 h and tanks were observed and photographed for 140 h in order to detect developing disease signs. At the end of each experiment, infectivity was calculated as the proportion of exposed fragments per tank that became infected. Both infected and non-infected fragments were crushed and individual CFU's were picked and transferred to fresh MA plates for further analyses and DNA extraction, as previously described. Bacterial strains causing disease signs in this experiment were given the simplified names: P1–P7 (P1–from *M. aequituberculata* in Nelly Bay GBR; P2 and P7 from *A. cytherea* in Majuro Atoll the Republic of the Marshall Islands; and P3–P6 from *P. speciosa* in Nikko Bay Palau) and were inoculated as pure cultures in the following experiments under these names. Bacterial strain P6 was isolated from seawater above diseased *P. speciosa* colonies at Nikko Bay Palau. It caused infections in a separate experiment and was therefore added to the list of putative pathogens. Strain P7 from the Marshall Island caused partial disease signs on only one *A. cytherea* fragment (out of four fragments) when inoculated into two tanks (n = 4 fragments per tank). Inoculation experiment I was repeated using this isolate (n = 12 fragments in each of 3 tanks) and it was removed from the putative pathogen list after failing to cause infections.

### Inoculation experiment II: Replicated exposure trial to fulfil Henle-Koch's postulates and test for virulence

To fulfil Henle-Koch's postulates, a large multi-replicated exposure trial using successful putative pathogens that initiated disease signs in inoculation experiment I were grown as pure cultures and inoculated (final inoculum concentration = 1×10^6^ cells ml^−1^) into multiple tanks with colony fragments (of *P. speciosa, M. aequituberculata A. hyacinthus*) collected from non-disease sites and acclimatized for 5 days. The number of fragments allocated to inoculation tanks at each site (n) was between 80–360, distributed as 4–6 fragments per tank, and the number of tanks per inoculation/control treatments (N) was between 4 and 12. At each site, 4 negative controls were run including: 1 treatment comprising tanks to which no bacteria were added, 2 treatments comprising tanks to which control bacterial strains were added at identical concentrations, and 1 treatment comprising tanks to which sterile bacterial media was added (1 ml MB per 1 L seawater) as potential “growth enhancer” for putative pathogens that might be already present on experimental coral fragments. Tanks were maintained temperatures identical to those measured at infection sites. Fragments in each tank were observed and photographed for the entire length of the experiment and developing disease signs were recorded. The experiments were terminated following mortality in infection tanks. Case mortality rate, or virulence (the proportion of infected fragments in each tank that died [36]) and the mean proportion of infected fragments per tank were calculated. Pathogenicity (the proportion of exposed fragments that died) was calculated following the formula of Thomas and Elkinton [35]: Pathogenicity = infectivity×virulence (where pathogenicity = # dying / # exposed, infectivity = # infected / # exposed and virulence = # dying / # infected). Finally, LT50 (the estimated time it takes to kill 50% of the infected fragments) was calculated as a temporal measure of virulence.

Both infected and non-infected fragments were crushed and streaked on agar plates to determine mean CFU's, as previously described. Total DNA was extracted from retrieved isolates for elucidation of taxonomic identity (16S rRNA gene sequence). Complete alignment (100%) of the 16SrRNA gene sequences retrieved from bacteria re-isolated from infected fragments and the 16S rRNA gene sequences of inoculated bacteria (P1–P6) allowed the fulfilment of Henle-Koch's postulates [20], namely, that:

1. An organism found only on infected corals could be isolated, taxonomically identified, and grown in pure culture.
2. The isolated organism reproduced disease signs when inoculated onto healthy corals.
3. An isolate retrieved from coral fragments that developed disease signs in inoculation experiments is demonstrated to be identical (by analysis of 16SrRNA partial gene sequences) with the organism used for inoculations.

### Other rules and criteria for supporting causality used by this study

Results of experiments and screenings conducted in this study were used to evaluate compliance with Evans' rules [21] and Hill's criteria [22], defined as alternative requirements for establishing disease causation. Both Evans' rules and Hill's criteria are listed in the Supporting Information section (see Materials and Methods S1).

### Adhesion of bacterial isolates to corals

To further test the physical ability of putative pathogens to migrate towards coral hosts, to adhere and to survive the initial contact with the coral host, before initial signs of infection are observed, mean bacterial CFU's were quantified from random sub samples of tank seawater (N = 4 seawater sub-samples per treatment, each taken from a different tank) following inoculation with the six coral pathogens (P1–P6) identified in Inoculation Experiment I (final inoculum concentration = 1×10^6^ cells ml^−1^). One ml of tank seawater was collected at inoculation time (t = 0 h), 1 h post inoculation (t = 1 h) and then at 12 h intervals (t = 12 h, t = 24 h, t = 36 h) from four infection tanks. 100 µL aliquots from each sample were spread in triplicates on agar plates containing Marine Agar and TCBS, as described previously. Mean CFU's ml^−1 ^g^−1^ wet weight were determined from three crushed coral samples per treatment at corresponding times [63]. In addition, control bacterial strains were also tested to determine if they adhered to coral fragments following inoculation into tanks. Finally, both putative pathogens and control bacteria were inoculated into four seawater tanks (per bacterial treatment) lacking coral fragments to test their ability to survive and remain suspended in the water column for the experiment's duration. Seawater samples were collected from these tanks and plated in triplicate as described previously to determine mean bacterial density in seawater (CFU's ml^−1^).

### The asocasein proteolytic Assay

The proteolytic activities of supernatants derived from 152 isolated bacterial strains retrieved by this study were tested by the asocasein assay based on a protocol by Windle and Kelleher [104]. Briefly, 1 ml of bacterial cultures grown to end logarithmic phase were centrifuged at 10,000g (Eppendorf 5415D centrifuge) for 5 min. Supernatant was removed and filtered through a 0.22 µm filter (Millipore, USA). 100 µL supernatant was incubated for 30min at 30°C with 5g ml^−1^ of asocasein as substrate (Sigma, USA) dissolved in Tris-Hcl (50 mM pH 8) containing 0.04% NaN_3_ (wt vol^−1^). The reaction was terminated by adding 10% (wt vol^−1^) of trichloroacetic acid (TCA) to a final concentration of 6.7% (wt vol^−1^) and incubating samples for 1 min. Samples were then centrifuged for 3 min at 10,000g and transferred to 700 µL of 525 mM NaOH. Absorbance of six replicates from triplicate culture samples was measured in 96 microtitre well plates at 450 nm using a Wallac spectrophotometer (Perkin Elmer, USA). Blank controls were prepared from supernatant derived from *E.coli* cultures boiled at 100°C for 10 min, treated with 5mg ml^−1^ asocasein and directly thereafter by TCA. Protease activity was calculated as proteolytic units, when 1U = 1000×(OD_450_ CFU^−1^)×10^9^ [64]. Isolates were divided into 3 groups based on their proteolytic activity: High activity (>3U), medium activity (1-3U) and no activity (<1U).

### Statistical analysis

Means and Standard Errors (SE) for bacterial colony forming unit (CFU) counts and for the proportion of infected colonies were compared among treatments using One-Way ANOVA (Statistica, StatSoft, Inc. USA). Colony forming unit (CFU) counts are presented in this study using logarithmic scales.

The association between categorical values related to bacterial isolates retrieved independently from diseased and non-diseased corals and demonstrating positive or negative proteolytic activity was estimated using 2×2 contingency tables (Pearson Chi-square). Significant results were determined when α≤0.05.

### Coral pathogens

Six coral pathogen strains that were identified by this study were submitted to the public collection of BCCM/LMG at the Gehnt University, Belgium and are available for acquisition under the following accession numbers: LMG23691-isolate P2 from a WS infected *Acropora cytherea* in Majuro Atoll the Republic of the Martshall Islands, LMG23692-isolate P5 from a WS infected *Pachyseris speciosa* in Nikko Bay Palau, LMG 23693-isolate P4 from a WS infected *P. speciosa* in Nikko Bay Palau, LMG 23694-isolate P6 from seawater above a WS infected *P. speciosa* in Nikko Bay Palau, LMG 23695-isolate P3 from a WS infected *P. speciosa* in Nikko Bay Palau and LMG 23696-isolate P1 from a WS infected *Montipora. aequituberculata* in Nelly Bay the GBR. 16S rRNA gene sequences of all coral pathogens identified by this study were submitted to Genbank (www.ncbi.nih.nlm.gov/Genbank/) under the following accession numbers: P1 (LMG23696)-EU372917, P2 (LMG23691)-EU372935, P3 (LMG23695)-EU372934, P4 (LMG23693)-EU372931, P5 (LMG23692)-EU372933, P6 (LMG23692)-EU372932. 16S rRNA gene sequences retrieved from isolates demonstrating positive results for proteolytic activity in both screening tests conducted by this study (asocasein assay and PCR amplification) were submitted to Genbank under the following accession numbers: EU372918-EU372930, EU372936-EU372939, and are presented in Fig. 7.

### Movie S2

*Acropora hyacinthus* fragments were inoculated with 1×10^6^ cells ml^−1^ of pathogen P2 (aquarium situated on the left hand side of the screen). Time lapse photography (every 20 seconds) was carried out for 36 h. Control treatment with no inoculation appears on the right hand side of the screen.

## Supporting Information

Materials and Methods S1 Evans' Rules and Hill's Criteria
(0.03 MB DOC)

Figure S1 Inoculation experiment I, Palau: A–B. *Pachyseris speciosa* coral fragments without inoculation (t = 0h and t = 96h). C–D. *P.speciosa* coral fragments inoculated with 1×10^6^ cells ml^−1^ of culture P3 (t = 0h and t = 96h). E–F. *P.speciosa* coral fragments inoculated with 1×10^6^ cells ml^−1^ of culture P4 (t = 0h and t = 96h). G–H. *P.speciosa* coral fragments inoculated with 1×10^6^ cells ml^−1^ of culture P5 (t = 0h and t = 96h).
(1.19 MB TIF)

Table S1 Adhesion Experiment
(0.03 MB DOC)

Table S2 Proteolytic activity of bacterial isolates (Nelly Bay GBR)
(0.04 MB DOC)

Table S3 Proteolytic activity of bacterial isolates (Palau)
(0.03 MB DOC)

Movie S1 WS outbreak in Majuro Atoll the Republic of the Marshall Islands (August 2004)
(10.34 MB AVI)

Movie S2 Time lapse inoculation experiment of *Acropora hyacinthus* fragments infected with 1×10^6^ cells ml^−1^ of pathogen P2 for 36 hours (aquarium situated on the left hand side of the screen). Control treatment with no inoculation appears on the right hand side of the screen.
(6.74 MB AVI)
