# Supplementary material for: Coral Pathogens Identified for White Syndrome (WS) Epizootics in the Indo-Pacific
Source: PLoS One. 2008 Jun 18;3(6):e2393. doi: 10.1371/journal.pone.0002393 (PMC2409975; doi:10.1371/journal.pone.0002393)
Supplement: Materials and Methods S1 — Evans' Rules and Hill's Criteria (0.03 MB DOC) [file pone.0002393.s001.doc]

**Material and Methods**

**Evans' Rules [21]:**

A. Prevalence of the disease should be significantly higher in those exposed to the putative cause than in cases controls not so exposed.

B. Exposure to the putative cause should be present more commonly in those with the disease than in controls without the disease when all risk factors are held constant.

C. Incidence of the disease should be significantly higher in those exposed to the putative cause than in those not so exposed as shown in prospective studies.

D. Temporally, the disease should follow exposure to the putative agent with a distribution of incubation periods on a bell shaped curve.

E. A spectrum of host responses should follow exposure to the putative agent along a logical biologic gradient from mild to severe.

F. A measurable host response following exposure to the putative cause should regularly appear in those lacking this before exposure (i.e., antibody, cancer cells) or should increase in a magnitude if present before exposure; this pattern should not occur in persons so exposed.

G. Experimental reproduction of the disease should occur in higher incidence in animals or man appropriately exposed to the putative cause than in those not so exposed; this exposure may be deliberate in volunteers, experimentally induced in the laboratory, or demonstrated in a controlled regulation of natural exposure.

H. Elimination or modification of the putative cause or of the vector carrying it should decrease the incidence of the disease (control of polluted water or smoke or removal of the specific agent).

I. Prevention or modification of the host's response on exposure to the putative cause should decrease or eliminate the disease (immunization, drug to lower cholesterol, specific lymphocyte transfer factor in cancer).

J. The whole thing should make biologic and epidemiologic sense.

**Hill’s criteria [22]:**

1. Strength of association: a strong positive statistical association between a factor

and disease

2. Consistency: If an association exists in a number of different circumstances,

then a causal relationship is probable

3. Specificity of association

4. Temporality of time sequence: Cause must precede effect.

5. Biological gradient: A dose response relationship between a factor and disease.

6-7. Plausibility and coherence: Compatibility with existing knowledge: It is more

reasonable to infer that a factor causes a disease if a plausible biological

mechanism has been identified.
