# Supplementary figures and images for: Coral Pathogens Identified for White Syndrome (WS) Epizootics in the Indo-Pacific
Source: PLoS One. 2008 Jun 18;3(6):e2393. doi: 10.1371/journal.pone.0002393 (PMC2409975; doi:10.1371/journal.pone.0002393)

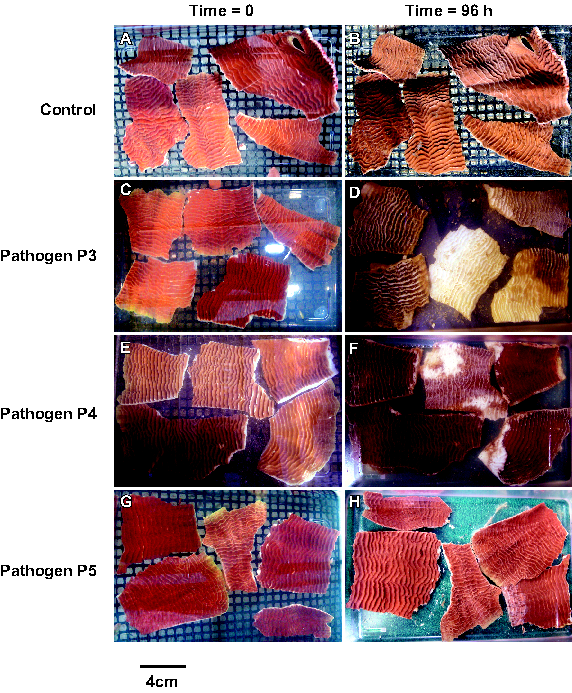

Supplement: Figure S1 — Inoculation experiment I, Palau: A–B. Pachyseris speciosa coral fragments without inoculation (t = 0h and t = 96h). C–D. P.speciosa coral fragments inoculated with 1×10^6^ cells ml−1 of culture P3 (t = 0h and t = 96h). E–F. P.speciosa coral fragments inoculated with 1×10^6^ cells ml−1 of culture P4 (t = 0h and t = 96h). G–H. P.speciosa coral fragments inoculated with 1×10^6^ cells ml−1 of culture P5 (t = 0h and t = 96h). (1.19 MB TIF) [file pone.0002393.s002.tif]
