# Supplementary material for: Coral Pathogens Identified for White Syndrome (WS) Epizootics in the Indo-Pacific
Source: PLoS One. 2008 Jun 18;3(6):e2393. doi: 10.1371/journal.pone.0002393 (PMC2409975; doi:10.1371/journal.pone.0002393)
Supplement: Table S1 — Adhesion Experiment (0.03 MB DOC) [file pone.0002393.s003.doc]

**Table S1: Adhesion experiment**

**^1^ Data for pathogens P3, P4, P5 and P6 was pooled**.

| **Outbreak** | **Nikko Bay Palau** | **Nelly Bay GBR** | **Majuro Atoll Marshall Islands** |
| --- | --- | --- | --- |
| Putative pathogens | P3-P6^1^ | P1 | P2 |
| Cultivable putative pathogens in SW at  t=12h (% from original inoculation at t= 0h) ^2^ | 13.5% | 12.0% | 13.0% |
| Cultivable putative pathogens on corals at  t=12h (factor calculated in relation to CFU’s at  t=0h) | 1049 x  Increase | 876 x  Increase | 484 x  Increase |
| Cultivable putative pathogens on corals at  t=12h (% from original inoculation at t=0h) ^3^ | 0.9% | 0.7% | 0.8% |
| % of cultivable putative pathogens missing from total original inoculation at t=12h ^4^ | 85.6% | 87.3% | 86.2% |

^2^ The percent (%) of cultivable putative pathogens in seawater at t=12h was calculated by the formula:

CFU’s ml^-1^ [t=12h] / CFU’s ml^-1^ [t=0h

^3^ The percent (%) of cultivable putative pathogen on corals was calculated by the formula:

Total CFU’s on coral fragments [t=12h] / Total original inoculation [t=0h].

^4^ The percent (%) of missing cultivable cells was calculated by the formula:

100 – (% CFU’s in sweater [t=12h] + % CFU’s on coral fragments [t 12h]).
