# Supplementary material for: Coral Pathogens Identified for White Syndrome (WS) Epizootics in the Indo-Pacific
Source: PLoS One. 2008 Jun 18;3(6):e2393. doi: 10.1371/journal.pone.0002393 (PMC2409975; doi:10.1371/journal.pone.0002393)
Supplement: Table S2 — Proteolytic activity of bacterial isolates (Nelly Bay GBR) (0.04 MB DOC) [file pone.0002393.s004.doc]

**Table S2:** **Proteolytic activity of bacterial isolates (Nelly Bay GBR)**

|  | **Bacterial isolates retrieved from diseased  *Montipora***  ***aequituberculata* fragments collected in the field^1^** | | |  |
| --- | --- | --- | --- | --- |
|  | **- ve Disease Signs^1^** | **+ ve Disease Signs^1^** | |  |
|  | **Healthy on diseased** | **Interphase** | **Skeleton** | **Total** |
| **+ ve PCR product ^2^** | 0 | 4 | 4 | 8 |
| **- ve PCR product ^2^** | 21 | 3 | 6 | 30 |
| **Total** | 21 | 7 | 10 | 38 |
| **High proteolytic activity^3^** | 2 | 4 | 5 | 11 |
| **Medium Proteolytic activity^4^** | 3 | 0 | 2 | 5 |
| **No proteolytic activity^5^** | 16 | 3 | 3 | 22 |
| **Total** | 21 | 7 | 10 | 38 |

^1^ Isolates retrieved from diseased *M. aequituberculata* field samples at Nelly Bay GBR: 1)

healthy fragment on diseased colony (H); 2) interface between lesion and healthy tissue (I);

3) exposed skeleton (S).

^2^ Specific amplification of *Vibrio* zinc-metalloprotease active zinc binding site.

^3^ High proteolytic activity >3U measured by the asocasein assay.

^4^ Medium proteolytic activity 1-3U measured by the asocasein assay.

^5^ No proteolytic activity <1U measured by the asocasein assay.
