# Supplementary material for: Coral Pathogens Identified for White Syndrome (WS) Epizootics in the Indo-Pacific
Source: PLoS One. 2008 Jun 18;3(6):e2393. doi: 10.1371/journal.pone.0002393 (PMC2409975; doi:10.1371/journal.pone.0002393)
Supplement: Table S3 — Proteolytic activity of bacterial isolates (Palau) (0.03 MB DOC) [file pone.0002393.s005.doc]

**Table S3: Proteolytic activity of bacterial isolates (Palau)**

|  | **Bacterial isolates retrieved from experimental**  ***Pachyseris speciosa* coral fragments^1^** | |  |
| --- | --- | --- | --- |
|  | **Infected colonies** | **Non-infected colonies** | **total** |
| **+ ve PCR product ^2^** | 27 | 2 | 29 |
| **- ve PCR product ^2^** | 19 | 10 | 29 |
| **Total** | 46 | 12 | 58 |

^1^ Bacterial Isolates retrieved from laboratory infected and non-infected *Pachyseris speciosa*

coral fragments sampled at the conclusion of inoculation experiment II.

^2^ Specific amplification of *Vibrio* zinc-metalloprotease active zinc binding site.
